# Binary classification of gynecological cancers based on ATR-FTIR spectroscopy and machine learning using urine samples

**DOI:** 10.1007/s10238-025-01684-1

**Published:** 2025-05-09

**Authors:** Francesco Vigo, Alessandra Tozzi, Flavio C. Lombardo, Muriel Eugster, Vasileios Kavvadias, Rahel Brogle, Julia Rigert, Viola Heinzelmann-Schwarz, Tilemachos Kavvadias

**Affiliations:** 1 Department of Biomedicine, University of Basel, Basel, Switzerland; 2 Department of Gynecology, Clinic for Gynecology and Gynecologic Oncology, University Hospital of Basel, Spitalstrasse 21, 4055 Basel, Switzerland; 3 University Hospital of Basel, Basel, Switzerland; 4 Medicine, University of Basel, Basel, Switzerland

**Keywords:** ATR-FTIR spectroscopy, Urine biomarkers, Gynecological cancers, Machine learning

## Abstract

Making an early diagnosis of cancer still in the early stages, when completely asymptomatic, is the challenge modern medicine has been setting for several decades. In gynecology, no effective screening has yet been found and approved for endometrial and ovarian cancer. Mammography is an effective screening method for Breast Cancer, as well as Pap Test for Cervical Cancer, but they are underused in third world countries because of their expensive and specific instrumentation. Previous studies showed how “machine learning analysis methods” of the spectral information obtained from dried urine samples could provide good accuracy in differentiation between healthy and ovarian or endometrial cancer. In this study, we also apply ATR-FTIR spectrometry’s practical, fast, and relatively inexpensive principles to liquid urine analysis from 309 patients undergoing surgical treatment for benign or malignant diseases (endometrium, breast, cervix, vulvar and ovarian cancer). The data obtained from those liquid samples were then analyzed to train a machine learning model to classify healthy VS cancer patients. We obtained an accuracy of > 91%, and we also identified discriminant wavelengths (2093, 1774 cm^−1^). These frequencies are close to already reported ones in other studies, indicating a possible association with tumor presence and/or progression.

**Supplementary Information:**

The online version contains supplementary material available at 10.1007/s10238-025-01684-1.

## Introduction

Early cancer detection is crucial for an effective treatment and is a determining factor for overall patients’ survival. Endometrial Cancer (EC) is the most common gynecological cancer in high-income countries and the fourth most common cancer in women [1]. Three-quarters of cases are diagnosed at an early stage (I or II) in which 5- and 10-year survival rates are 95% and 77%, respectively, but when diagnosed at a late stage (IV), the survival is poor with only 14% of women surviving for 5 years [2]. Ovarian Cancer (OC) is the most lethal gynecological cancer, late-stage presentation has a 5-year survival rate of 29%, in contrast to 92% for early stage disease. Unfortunately, about 75% of patients are diagnosed at an advanced stage because of its asymptomatic nature [3]. Today there is no established screening tool at our disposal for EC and OC: Sex steroid hormones, L1CAM, adiponectin, hysteroscopy, and biopsy for EC as well as pelvic ultrasonography and serum CA-125 for OC have been proposed and used in various research settings, but they have been proved to be either too invasive and of low cost-to-benefit ratio, or too ineffective and inaccurate [4, 5].

Even when available, for example in cervical (CC) or breast cancer (BC), the contemporary screening tools are expensive and require highly qualified infrastructure and personnel, making access to them difficult or even impossible, mostly in developing countries [6, 7]. To overcome those limits, one of the latest and most rapidly emerging diagnostic platforms in medicine is liquid biopsy. The term was introduced by Pantel and Alix-Panabières to identify circulating tumor cells (CTCs) in patients’ blood. This approach was firstly pursued as an early detection method, and its use was extended to the assessment of benign or malignant disease in blood as well as in other body fluids such as urine, saliva, cerebrospinal fluid (CSF), or pleural effusion [8, 9]. Assuming that the development of tumors can generate detectable traces, since most of the metabolites can be found in urine, it is possible, analyzing them, to obtain indirect information about the pathological metabolism of several organs as well as any inflammatory or neoplastic processes. Urine analysis gained particular interest since its collection is easy, non-invasive, and familiar to the patient [10, 11].

An analytical method particularly suitable to detect those potential markers is the vibrational spectroscopic technique, which in the last two decades, raised scientific interest due to its rapid, non-destructive, and relatively inexpensive characteristics. Its potential has already been shown in different bio-fluids, such as blood, plasma, saliva, and dried urine; when coupled with statistical analysis and machine learning algorithms, such as random forest, the analysis, and classification of the obtained spectra, could reach classification power to an extent that was formerly not possible [12–15].

To date, there are a handful of studies that have examined the potential of urine as a rapid and non-invasive method for the detection of gynecological cancers. All of them used dried or pretreated samples in order to avoid the excessive absorption of infrared radiation through water. The aim of this study is to examine the potential of spectral analysis using machine learning algorithms in urine with minimal processing and in a liquid state, paving the way for a potentially fast and accurate patient screening method.

## Material and methods

### Study population and sample collection

Data collection and analysis were performed according to the local regulations and guidelines after approval of the local ethical committee (*ID 2022–00109*). The urine samples were collected and used after obtaining written informed consent from all patients according to the General Consent for using health-related data and samples for research purposes, following the Swiss legal regulations [16].

From the patients’ administration registry of the hospital, we reviewed all gynecologic patients who were admitted to our clinic between January 2015 and July 2020 and underwent major gynecologic surgery. Then, we selected urine samples from patients who fulfilled all of the following criteria: (i)18 years and older, (ii) signed informed consent of the above-mentioned General Consent, (iii) hysterectomy and/or salpingo-oophorectomy, as well as Breast surgery with histological confirmation of the diagnosis. Firstly, the patients with gynecological cancer were selected, and consecutively a matched control group of patients with benign gynecologic conditions was formed in a 2/1 ratio (benign/malignant).

Urine samples were collected and banked before surgery: during the outpatient routine pre-surgery workup, during their hospitalization the day before the intervention, or just before surgery in the operating theater. Samples were stored in suitable plastic cryotubes in a deep freezer at − 80 °C, with 24/7 automated monitoring to avoid temperature fluctuations. Before analysis, all samples were left to thaw at room temperature, and, after manual shaking 20 μL were deposited on ATR (attenuated total reflection) crystal without any other pre-treatment except for a vigorous mechanical shaking (vortex or by hand).

### Spectroscopic sample acquisition

The spectroscopic analysis was performed using 20 μL of the urine sample at room temperature, which was deposited directly in liquid form on the ATR crystal using a standard 2–200 μL pipette. Each spectrum acquired was the average of 24 consecutive measurements on the same sample. This way, the various noise sources (e.g., instrumental, systematic, random, and temperature fluctuations) are reduced, the reproducibility of the measurements is better estimated, and the overall performance of the method is increased. For each patient, 3 samples were measured, and each sample retrieved 24 scans (72 scans in total for a total sample’s consumption of 60 μL urine), to exclude any possible casual interference or alteration. The instrument was equipped with a single reflection ATR-FTIR (Platinum-ALPHA, Brucker, Germany) diamond crystal. Spectra were measured at 4 cm^−1^ spectral resolution, averaging 24 interferograms (40 s). The wavelength range was 4000–400 cm^−1^. Before every measurement, the instrument’s accuracy was tested using a propanol solution, with a known spectral characteristic. A background spectrum of the clean empty cell was acquired every ten sample measurements, to capture the dark noise of the instrument. The crystal was cleaned with pure water and paper towel after every measurement. A spectrum of double distilled water was also obtained with the same procedure and later used in the analysis process to reduce the noise. The spectrometer was controlled using OPUS 7.0 software from Brucker Optik GmbH ® (Ettlingen, Germany). The total procedure time (excluding thaw time, 5–10 Minutes ca) was approximately 1 min.

In the context of this study, 309 urine samples were collected, of which 206 from healthy individuals (control group with benign gynecologic conditions) and 103 from cancer patients (29 samples from patients with breast cancer, 32 from endometrial cancer, 31 from ovarian cancer, including borderline, phallopian tube and peritoneal cancer, 10 from cervical cancer and 1 vulvar cancer (Table 1).

**Table 1:** Patient cohort demographics, clinical and tumor characteristics

	Breast Carcinoma (*N* = 29)	Cervical Carcinoma (*N* = 10)	Ovarina Carcinoma (*N* = 31)	Uterine Carcinoma (*N* = 32)	Vulva Carcinoma (*N* = 1)
Age^a^	46 (41,58)	47 (44,54)	63 (53, 71)	55(49,75)	80 (80,80)
BMI^a^	24 (22,30)	27 (19,32)	23 (20,26)	26 (24,31)	35 (35,35)
Ethnicity					
Caucasian	28 (96.6%)	10 (100%)	30 (96.8%)	31 (96.9%)	0 (0%)
Black	1 (3.4%)	0 (0%)	0 (0%)	1 (3.1%)	0 (0%)
Asian	0 (0%)	0 (0%)	1 (3.2%)	0 (0%)	0 (0%)
Tumor Grade					
1	3 (11%)	0 (0%)	2 (8%)	12 (36%)	NA
2	13 (48%)	4 (44%)	0 (0%)	10 (32%)	NA
3	11 (41%)	5 (56%)	23 (92%)	10 (32%)	NA
Stage					
I	9 (32%)	3 (30%)	6 (21%)	18 (75%)	0 (0%)
II	8 (29%)	3 (30%)	2 (7%)	4 (17%)	0 (0%)
III	5 (18%)	0 (0%)	12 (43%)	1 (4%)	1 (100%)
IV	6 (21%)	4 (40%)	8 (29%)	1 (4%)	0 (0%)
Menopausal	9 (32%)	5 (50%)	23 (79%)	20 (63%)	1 (100%)

For breast cancer, the AJCC staging was used

Patient characteristics and diagnoses [^a^Median (IQR)] Staging for breast cancer according to the American Joint Committee on Cancer (AJCC), all other according to the International Federation of Gynecology and Obstetrics (FIGO)

### Histopathological assessment

All postoperative surgical specimens were treated with formalin fixation and paraffin embedding for histological examination. All benign and malignant specimens were analyzed by a consultant gynecological pathologist, according to the hospital’s internal protocols. Staging of cancers was performed according to the International Federation of Gynecology and Obstetrics (FIGO) systems for endometrial, ovarian, cervical, and vulvar cancers, and according to the American Joint Committee on Cancer (AJCC) systems for breast cancers [17, 18].

### Computational analysis

The samples were read from.dpt files and loaded into Python v3.10.5. The triplicated spectra for each patient were averaged and subtracted from double distilled H_2_O spectra obtained in a similar environmental condition as the urine samples.

**Fig. 1:**
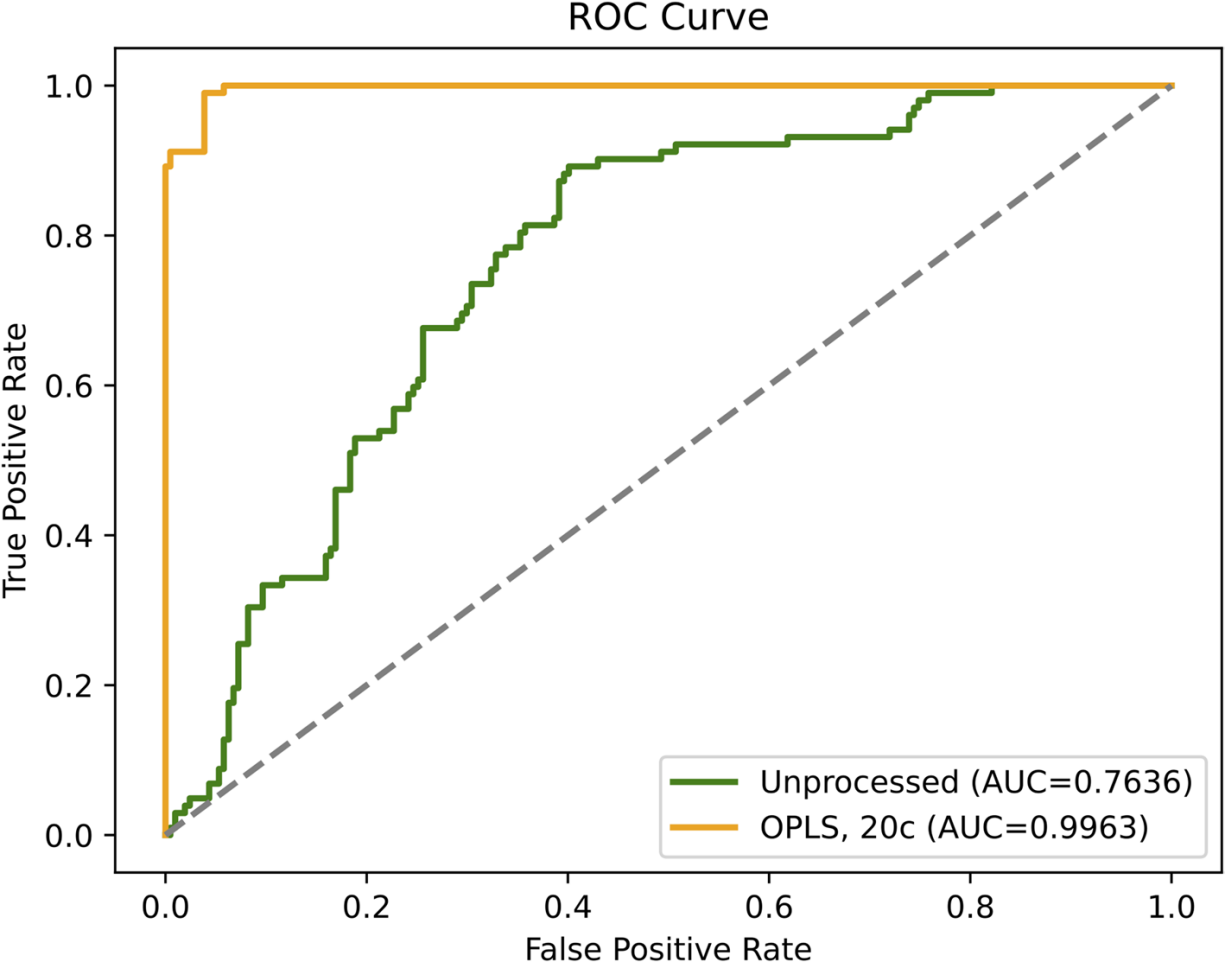
ROC curve indicates the accuracy of prediction of the PLS regression model using OPLS preprocessed data or not. The OPLS using 20 components appears to increase the metric scores of the predictions (cancer and benign gynecologic conditions)

We applied the Savitzky–Golay filter, a digital filter that can be applied to a set of digital data points to smooth the data, increasing its precision without distorting the signal tendency. We applied ten passages, the first derivative with a second-degree polynomial order for baseline correction. Spectra were then normalized by standard normal variate (SNV) before clustering analysis, and Orthogonal projection of latent structures (OPLS) for the supervised analysis with 20 orthogonal components. OPLS data transformation was then included, to improve feature selection by removing variations in the spectral data that were not correlated with the target variable (presence or absence of cancer). The classification power of the OPLS transformed data was assessed through Partial Least Squares (PLS) regression. The evaluation metrics included in the code, such as R2 (coefficient of determination), accuracy, and AUC (Area Under the ROC Curve) (Fig. 1). Python v3.10.5 was used within a conda-based virtual environment in conjunction with scikit-learn library v1.2.1, pandas 1.4.3, NumPy v1.23. Because of the class imbalance, we performed a tenfold cross-validation in the GridSearchCV for the model. To ensure reproducibility and address class imbalance, we implemented multiple strategies: (1) using a fixed random seed (333) for all random operations, (2) applying stratified sampling during train/test splitting to maintain class distribution, and (3) utilizing class weights inversely proportional to class frequencies.

In the analysis, we encoded the cancer patients as 1 and the control group as 0. We therefore used these encodings to perform supervised classification based on random forest embedding. Algorithm embedding can perform accurate predictions and be less biased toward overfitting than other machine learning techniques, and its output can be easily interpreted. To apply the algorithm to our data, we conventionally split the dataset into 30% testing and 70% training. We performed a hyperparameter optimization for a RandomForestClassifier using GridSearchCV to find the best model parameters (Supplementary Figs. 4 and 5). The optimal parameters identified are as follows: criterion set to either Gini or entropy, 21 estimators, maximum tree depth of 4, a minimum number of samples required to split a node at 8, and a minimum leaf sample size of 2. We also used a tenfold cross-validation on our dataset to test the model’s capacity and to observe eventual bias in the data.

### Statistical analysis

All statistical analyses were conducted using technical triplicates (309 samples) for each spectroscopic reading. All spectra data were the results of an average between 24 different recordings. We assessed the data distribution using the Shapiro–Wilk, resulting in non-normality of the data. For direct spectra comparison we used a nonparametric test: Kruskal–Wallis one-way analysis of variance with an alpha of 0·05, using Python 3.10.5 and statsmodels with statannotation and scipy.stats. For the tables, we used R version 4.2.2 using gtsummary and gttable. Model performance evaluation was based on the following metrics: precision, recall, and F1 Score were obtained by scikit-learn python library “classification report.”

Precision is the ratio of correctly predicted positive observations to the total predicted positives. It is defined as:

$${\text{Precision}} = \left( {{\text{TP}}} \right)/\left( {{\text{FP}} + {\text{TP}}} \right)$$

Recall is the ratio of correctly predicted positive observations to all observations in actual class. It is defined as:

$${\text{Recall}} = \left( {{\text{TP}}} \right)/\left( {{\text{TP}} + {\text{FN}}} \right)$$

The F1-score is the harmonic mean of precision and recall. It is indicated when the target class distribution is imbalanced. The F1-score is defined as:

$${\text{F1}} - {\text{score}} = {2}*\left( {{\text{Precision}} \times {\text{Recall}}} \right)/\left( {{\text{Precision}} + {\text{Recall}}} \right)$$

Accuracy is defined as the ratio of the correct classifications to the number of total classifications

$${\text{Accuracy}} = \, \left( {{\text{TP }} + {\text{ TN}}} \right)/\left( {{\text{TP }} + {\text{ TN }} + {\text{ FP }} + {\text{ FN}}} \right)$$

Sensitivity and specificity were calculated by standard definitions [sensitivity = (TP)/(TP + FN), specificity = (TN)/(TN + FP)] (whereas TP = true positive, TN = true negative, FP = false positive, FN = false negative).

## Results

**Fig. 2:**
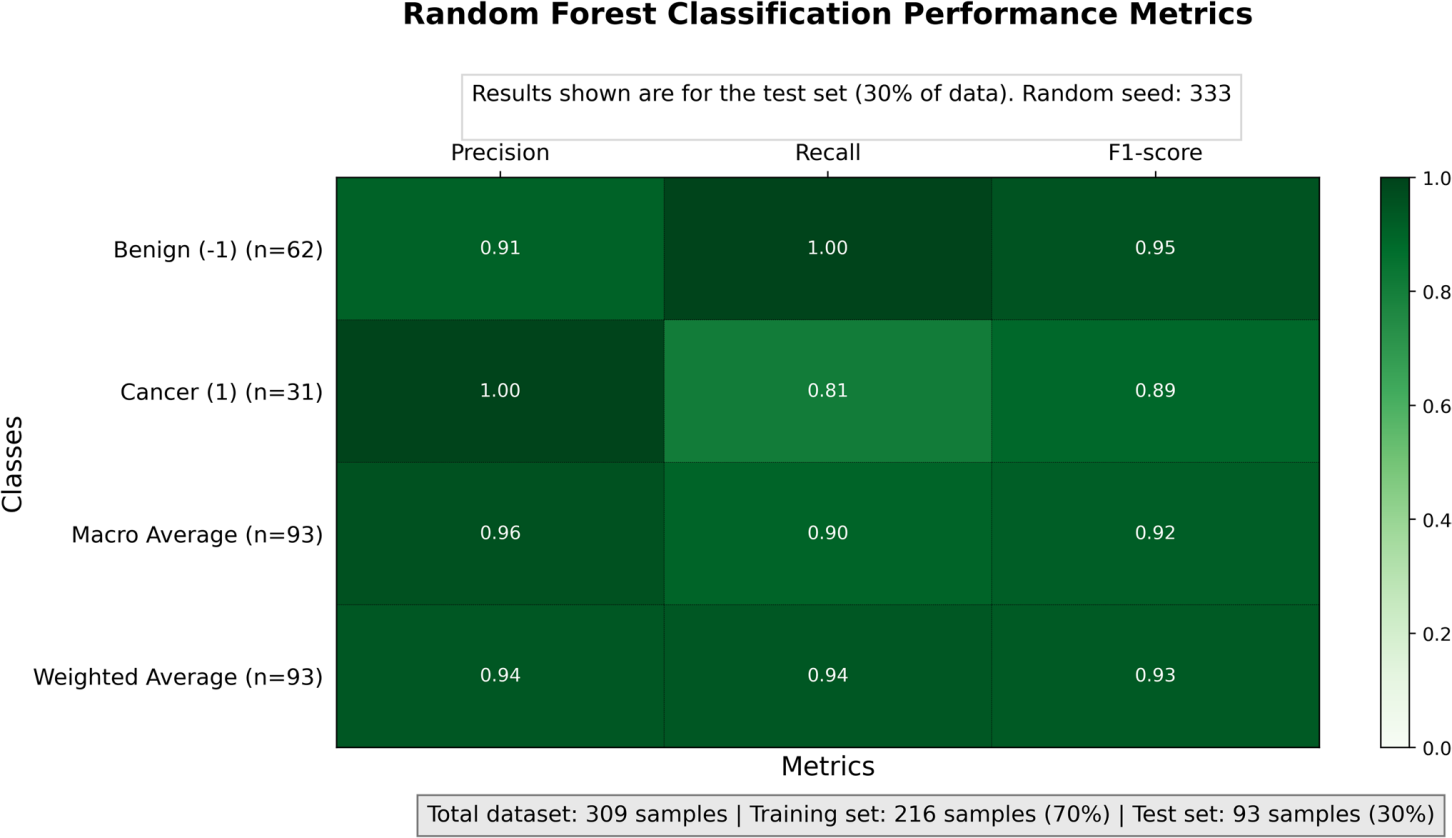
Confusion Matrix, the table indicates the method performance in the test dataset. Here precision, Recall, and F1-score are shown. In the table, the values taken into account data unbalanced between tumor vs normal samples

Using the random forest classification as described above on the denoised data, we obtained a precision of 91%, in which samples from the control group were never misclassified. We used metrics such as F1-score and ROC curves to characterize the quality of the model, keeping also into account that our dataset was unbalanced for most of the classes. As F1-score, we obtained 95% for the control group and 89% for true cancer samples (Fig. 2) with a weighted global F1-score of 93%.

To validate the discriminative power of the identified frequencies, we used the most salient frequencies pinpointed by the Random Forest (RF) ensemble on the OPLS transformed data, as determined through the SHapley Additive exPlanations (SHAP) Python library, to categorize the sample accurately. Subsequently, we further investigated those frequencies to subset the water baseline subtracted data and we found that most of the frequencies are statistically different between the cancer versus the healthy samples.

**Fig. 3:**
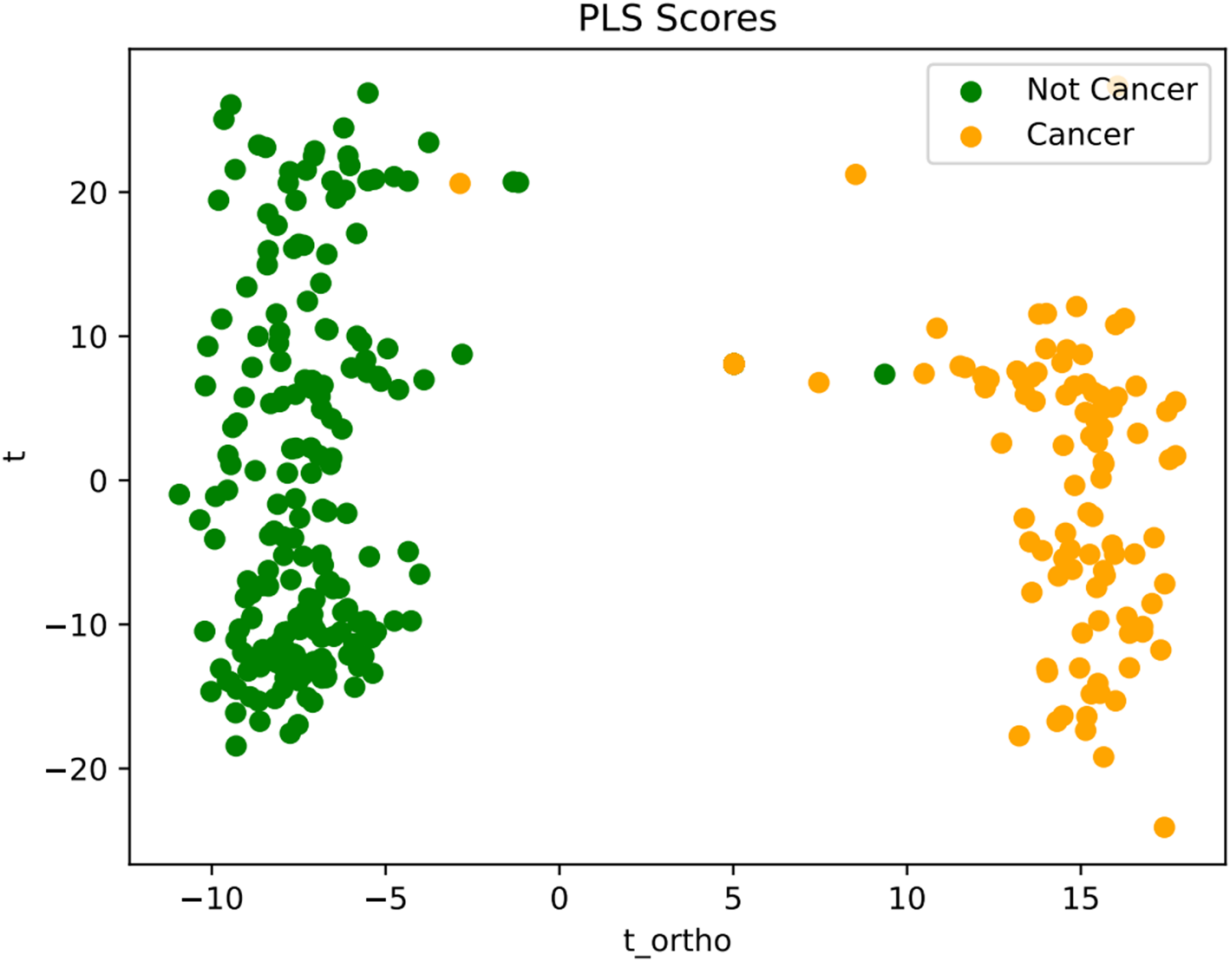
Visualization of PLS Scores Derived from OPLS Transformed Spectral Data for Cancer Diagnosis. This figure displays the first two PLS scores resulting from the application of PLS regression to the spectral data, which was preprocessed using OPLS with 20 orthogonal components to enhance feature selection. The scores illustrate the effective separation between cancerous (represented in yellow) and benign gynecologic conditions (represented in green) samples, highlighting the discriminative power of the selected features in distinguishing between the two classes

**Fig. 4:**
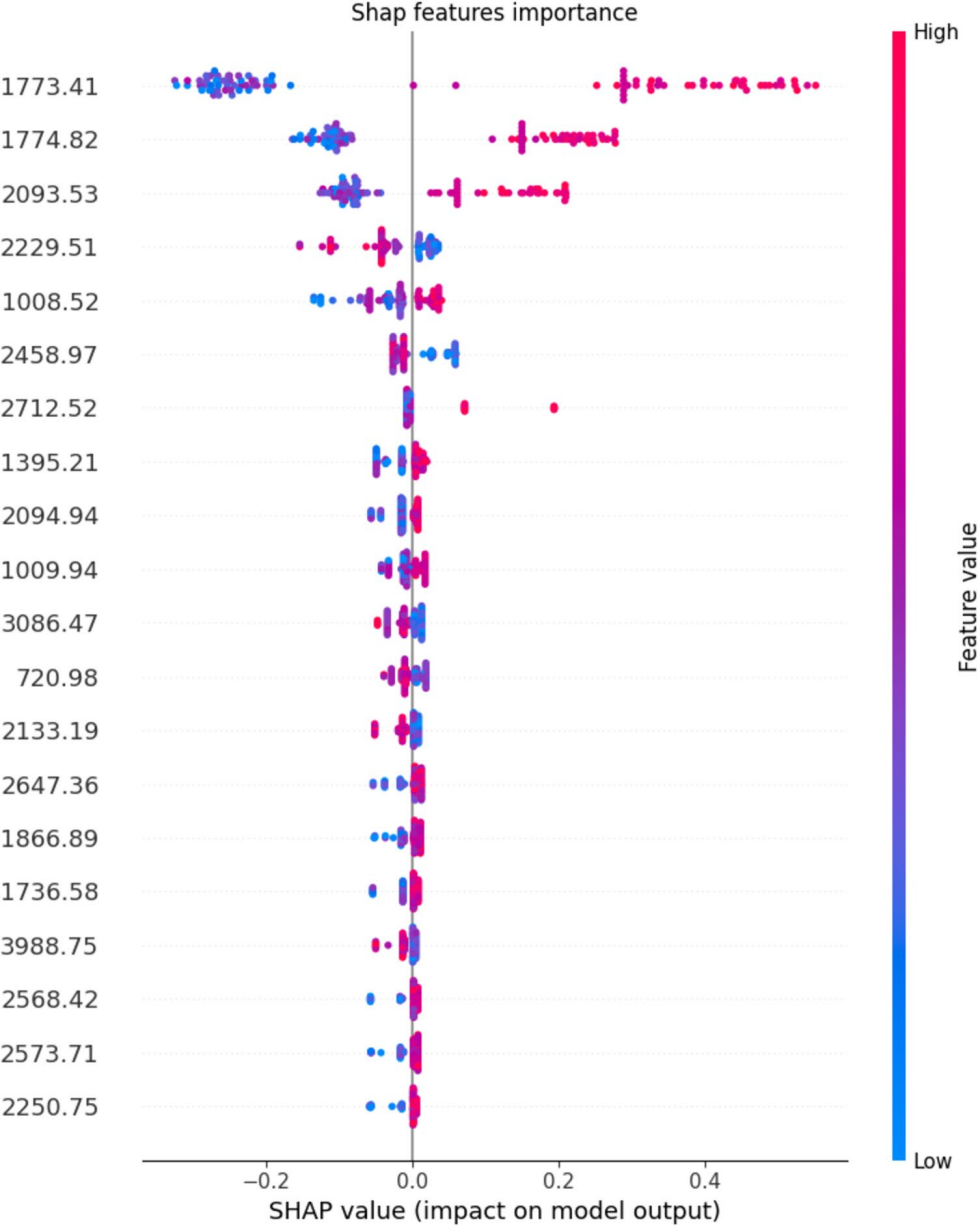
Top 20 SHAP features importance. The important features are marked in red and go to the right part of the plot directionality

**Fig. 5:**
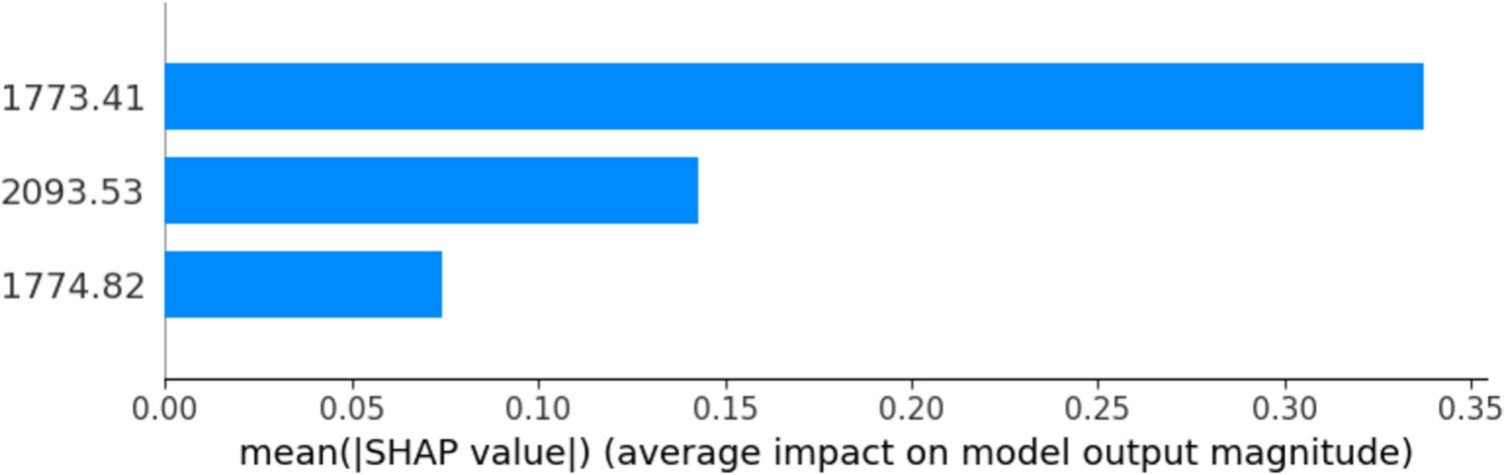
Top 3 frequencies as SHAP features importance in the developed Model. On the x axes, the average impact on RF model output magnitude is reported. This effectively indicated which one of the frequencies are prominently guiding the classification model

The distinction between control group and cancer samples is clearly depicted in the visualization of PLS regression scores following OPLS data transformation (Fig. 3). We then used the SHAP values to define the most relevant parameters obtained by the model since the algorithm can show how much each predictor contributes to the RF classification, either positively or negatively, to the target variable (Fig. 4). We identified 3 specific frequencies that provided enough information to separate the samples controls from the tumor (Fig. 5). Because of the proximity of 1773 and 1774 cm^−1^ we included only the peak at 1773 in our analysis.

We observed that the predictor frequency 1773 cm^−1^ positively contributed to the model. They both could be associated with Carbonyl (C=O) stretching and Amide I band. A similar pattern was also observed in frequency 2093 cm^−1^, associated with Cyanide (C≡N) stretching and secondary and tertiary amine N–H stretch. The other predictors had a lower contribution to the model, but taken together, they increased the overall model quality.

**Fig. 6:**
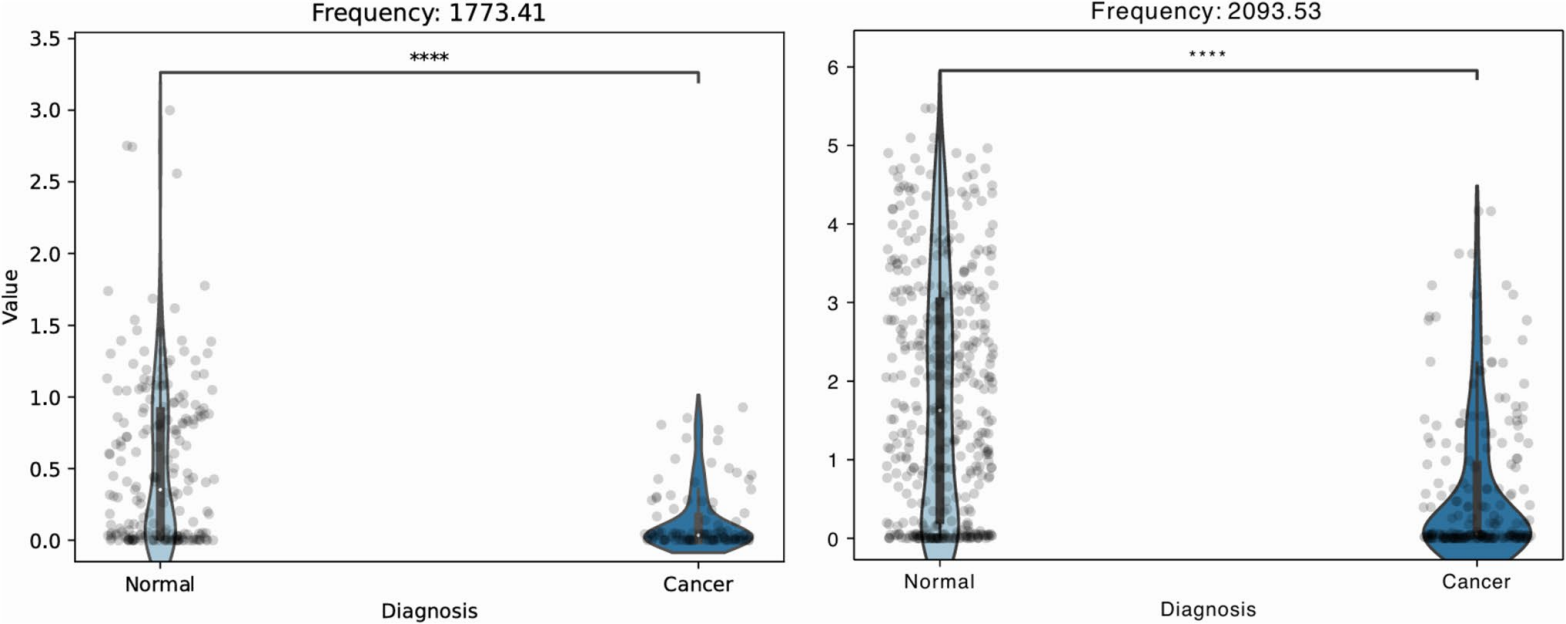
Binarized patients disease status in control group (*N* = 206) and cancer (*N* = 103). The scatter plots and the violin plots indicate the overall spread of the absorbance detected in the various samples. The plots indicate high significance (based on Kruskal–Wallis one-way analysis of variance with an alpha of 0·05) between the cancer samples and the normal samples

Of these frequencies, many were higher in the control group than in cancer. We performed a nonparametric Kruskal–Wallis one-way analysis of variance on these frequencies to find potentially more robust ones. From our data, it seems that the frequencies at 1773 and 2093 cm^−1^ best separate between the samples (Fig. 6).

### Spectral biomarkers

The developed model highlights how some specific frequencies are particularly important and capable of determining correct classification between control group and cancer, and they are decisive with regard to computation in machine learning.

The interpretation of absorption bands in an Attenuated Total Reflectance Fourier Transform Infrared (ATR-FTIR) spectrum of a complex biological fluid such as urine necessitates not only an understanding of the potential chemical constituents but also a holistic examination of the entire spectral landscape. The multivarious organic and inorganic compounds present in urine can give rise to numerous vibrational modes, rendering spectral deconvolution challenging [19].

Given this context, an absorption band at 1773 cm^−1^ which was our main finding could potentially be attributed to a couple of vibrational modes:

1.1 Carbonyl (C=O) stretching: Vibrations in this range may suggest the presence of compounds such as ketones, esters, or carboxylic acids, where carbonyl functional groups are common, particularly in lipids [20].

1.2 Amide I band: This spectral feature primarily stems from C=O stretching vibrations in proteins [21]. These could be indicative of altered protein metabolism or the presence of specific proteins or peptides that are markers for cancer. Alterations in protein expression have been widely studied in gynecological cancers [22, 23].

For the absorption band observed at approximately 2093 cm^−1^ within the mid-infrared region, several molecular vibrations might be considered:

2.1. Cyanide (C≡N) stretching: This range is typical for the vibrational mode of the cyano functional group [21, 24].

2.2 Secondary and tertiary amine N–H stretch: These could reflect changes in nitrogen metabolism, possibly relating to the increased protein turnover or altered nucleotide metabolism often associated with cancer [21–25].

## Discussion

Comparing our results with those obtained by other authors, we screened the available literature. The achievements obtained in the spectroscopic field applied to the research of oncological pathologies are scarce. Only two articles applied those techniques to screen gynecological cancers. In 2018, Paraskevaidi et al. published a study with a small group of patients (10 endometrial cancer, 10 ovarian cancer, and 10 controls), performing infrared analysis on air dried urine samples. They analyzed the preprocessed spectra of urine using as classification methods the Partial least squares discriminant analysis (PLS-DA), the principal component analysis with support vector machines (PCA-SVM) and genetic algorithm with linear discriminant analysis (GA-LDA). The frequencies they obtained for the classification of endometrial cancer were 1593 cm^−1^, 1508 cm^−1^, 1462 cm^−1^, 1400 cm^−1^, 1335 cm^−1^, 1041 cm^−1^. Two of the six discriminatory peaks were lower in cancer cases. These were attributed to the C–C vibrations of the phenyl rings of proteins (1593 cm^−1^, Amide II) and CH2 vibrations of lipids (1462 cm-1). The other IR-bands were mostly associated with proteins and nucleic acids. The frequencies obtained for ovarian cancer were 1597 cm^−1^, 1508 cm^−1^, 1408 cm^−1^, 1373 cm^−1^, 1231 cm^−1^, 1041 cm^−1^. The increase in discriminatory peaks, attributed to proteins and nucleic acids, was also mainly observed in the cancerous samples, except for the 1597 cm^−1^ peak assigned to the C–C phenyl ring of proteins [26]. The second available paper was published in 2022 by Ramirez et al., from the same research group. They compared 109 urine samples solely of endometrial cancer patients to 110 samples from healthy individuals. The authors report a sensitivity of 98% and a specificity of 97%. Spectra acquisition was also provided using an ATR-FTIR spectroscopy from dried at room temperature Urine. The OPLS transformed spectra were analyzed using then PLS-DA. The most relevant frequencies were those occupying the phenyl ring vibrations, CH out of plane vibrations, and deformations attributed to aromatic molecules, as 1585 cm^−1^ (phenyl ring deformation), 1485 cm^−1^ (CH deformation), 810 cm^−1^ (ring CH deformation), between 628 and 786 cm^−1^ (CH out of plane bending vibration), 609 cm^−1^ (ring deformation of phenyl), and 520 cm^−1^ (Cα = Cα’ torsion and ring torsion of phenyl). The peaks corresponding to lipids, between 1762 and 1797 cm^−1^ (C=O, C=C stretching) were a common marker in all the comparisons made, as well protein markers in Amide I band as 1662 cm^−1^ [27]. The fact that similar bands may be over- or under-expressed should not detract from the validity of these results. Indeed, it is well-known and demonstrated how different substances and biomarkers can potentially be expressed differently and variably, due to both type, subtype and maybe stage of disease [28].

Failing to find a succession to the encouraging results obtained by Paraskevaidis and Ramirez, but convinced of the potential of these techniques and driven by the need to find new ways for cancer screening in the gynecologic setting, we investigated further to verify and improve those previous results. Firstly, we expanded the investigation collecting urine samples from numerous oncological patients, continuing the collection and reaching more than 100 units. The control group of the study is then composed of more than 200 patients with benign diagnoses, also verified histologically for at least one of the searched tumors, besides clinical and intraoperative visual control. This is the first study in the field that presents a relatively large amount of samples (N 309). Secondly, before starting with the measurements, we screened all the spectroscopic techniques used in the literature, finding mostly laborious extracting methods, complicated procedures, and no direct liquid analysis [14]. So we decided to try one of the easiest, less expensive spectroscopic devices, the ATR-FTIR, using directly liquid samples without any previous processing of the sample. We were aware of the fact that using liquid specimens, some of the hydrated compounds could provide different readings as when the urine samples are read in dried conditions, and that IR water absorbance could hide and withhold important information. However, we focused on the most straightforward, cost-efficient, and simple possible application of this method, obtaining sufficient data for robust analyses and concrete results. Infect we reached an accuracy of 91% a very encouraging outcome, suggesting that there is potential in this method.

Nevertheless, it is remarkable, or at least interesting, that despite the use of not entirely similar techniques and methods (which makes direct comparison difficult), some of our results are similar to those previously published. Among the several peaks (Supplementary Fig. 1) identified as the major responders in the model we developed, the three most significant frequencies were consistently more expressed in the control group and less in cancer samples. As previously reported, these frequencies fall within the amide I band and amine stretch, as also reported by Ramirez and Paraskevaidi. Numerous papers report alterations in protein, or tumor tissue metabolism that could account for these alterations, thus supporting the quality of our findings, even if they are generic [21, 22]. The capability to ascertain the absence of disease using a spectroscopic marker renders this approach particularly suitable not only for screening purposes but also for follow-up of tumors with a high propensity for recurrence, such as gynecological cancers. The complexity of the analyzed medium, the urine, the possible variables as well as the interferences that the substances may have, make the analysis of the obtained spectra (sum of the spectra of all chemical species in the fluid) extremely difficult. A deep understanding of the sample’s composition, comparative study with reference spectra, comprehensive consideration of other spectral features, and so forth, are vital for the accurate identification of chemical constituents in a urine sample using ATR-FTIR spectroscopy. While a very rich source of information can be found in the urine spectrum, being able to trace a single molecule/biomarker responsible for the detection of tumor presence is at this time a far and future goal.

Even though the potential of spectroscopic methods has been known for a long time, relatively few studies have been directed to clinical application. The studies carried out to date vary in methods and types of patients and diseases to which they have been applied. They have also consistently reported small sample sizes, presenting mostly insignificant results, impossible to apply to larger samples [14]. The number of specimens ideally should be in the range of thousands, even millions, and should be collected by multiple and independent researchers, from different populations and by using different laboratory instrumentation.

Only after these steps can one reliably draw conclusions about the objective accuracy, sensitivity, and specificity of the method, as well as address key questions regarding the data, such as which classification algorithm yields the best performance and whether specific spectral bands can be identified as potential biomarkers.

## Conclusion

This study confirms the potential of the mid-infrared spectroscopy in the detection of gynecological cancers from urine samples reaching an accuracy of 91%, an F1-Score of 95% for healthy samples and 89% for true cancer samples, respectively. A pool of potential spectroscopical biomarkers, on which our classification model is based, was identified, especially for the peak 1773 cm^−1^, suggesting the possible presence of proteins or lipids target.

The simplicity of the method in terms of rapidness, non-invasiveness, and cost-effectiveness with the ability to be performed by non-specialized paramedical personnel makes this test potentially suitable not only for clinical routine practice, early detection, and monitoring of the disease but also an invaluable tool for screening the population worldwide. Ideally, the goal is not only to be able to detect cancerous patients by extracting a spectroscopic pattern in the urine samples but also to identify the type of each cancer further. As discussed, the current dataset is quite imbalanced to perform such a task, and this is planned for as a future work. Also, Integrating spectroscopic data with urine chemical–physical parameters and other patient data (clinical examination, medical history, previous histological diagnoses, etc.) could help to get more robust results despite the relatively low number of samples. In the presence of additional samples, one could also study whether there is a relation between the presence of a specific spectra and a patient response to treatments, this would be invaluable information to stratify patients.

Although spectrochemical methods have solid advantages and potential in the clinical domain, they still remain underutilized. One of the primary reasons is the need for more standardization of the sample collection and data processing methods. The impact of variations between different operators, equipment, laboratories, and populations is not yet thoroughly investigated, but very much needed in order to establish clinical protocols for sample retrieval, handling, and measurement.

In order to prove the accuracy, performance and the generalizability of the model, further tests and rigorous clinical trials, prospective and double blind studies should be performed. With this study, we can show valuable data on using liquid urine samples directly for spectroscopic analysis for potential patient stratification and biomarkers discovery.

## Supplementary Information

Below is the link to the electronic supplementary material.

In this column plot, are depicted all the frequencies that appeared to be relevant in the random forest model. (PNG 166 kb)

PCA analysis of water removed spectra without additional data preprocessing, filtering the 46 frequencies found to be relevant from the RF classifier. (TIF 15625 kb)

Learning curve, to evaluate the performance of our model. The curve demonstrates the relationship between the training size and the model’s accuracy. It shows how the model’s performance improves as the size of the training dataset increases. The training score (in red) indicates good performance of the model, the green curve with the confidence interval shows how the model could benefit from additional unseen data. Both curves appear to converge with more training examples, indicating diminishing model improvement from additional data. The increased data fed to the model shows a reduced score, indicating that our model is not overfitting. (TIF 10800 kb)

We used a random seed (333) for the train/split and we handled the class imbalance using class weighting. The stability of our feature importance interpretations was assessed by analyzing SHAP value distributions across multiple independent model runs with different random seeds. As shown in the figure, the top features maintained consistent importance rankings across runs, with the most influential features (feature1 and feature2 and feature3) showing strong stability. This analysis confirms that our model's interpretations are robust to variations in data sampling and model initialization, strengthening confidence in the identified biomarkers. (PNG 24 kb)

For model selection, we compared Random Forest against Logistic Regression, Support Vector Machines (SVM), and Gradient Boosting using 5-fold cross-validation. As shown in Figure below, all models achieved high performance metrics, with Logistic Regression slightly outperforming others across accuracy (96%), precision (96%), recall (96%), and F1 (96%). However, we selected Random Forest for our final analysis due to its strong performance combined with its interpretability advantages through built-in feature importance measures and compatibility with SHAP for detailed feature attribution. (PNG 25 kb)

## Data Availability

Data available upon request due to restrictions from the local regulatory authorities.
