# Supplementary figures and images for: Binary classification of gynecological cancers based on ATR-FTIR spectroscopy and machine learning using urine samples
Source: Clin Exp Med. 2025 May 9;25(1):143. doi: 10.1007/s10238-025-01684-1 (PMC12064457; doi:10.1007/s10238-025-01684-1)

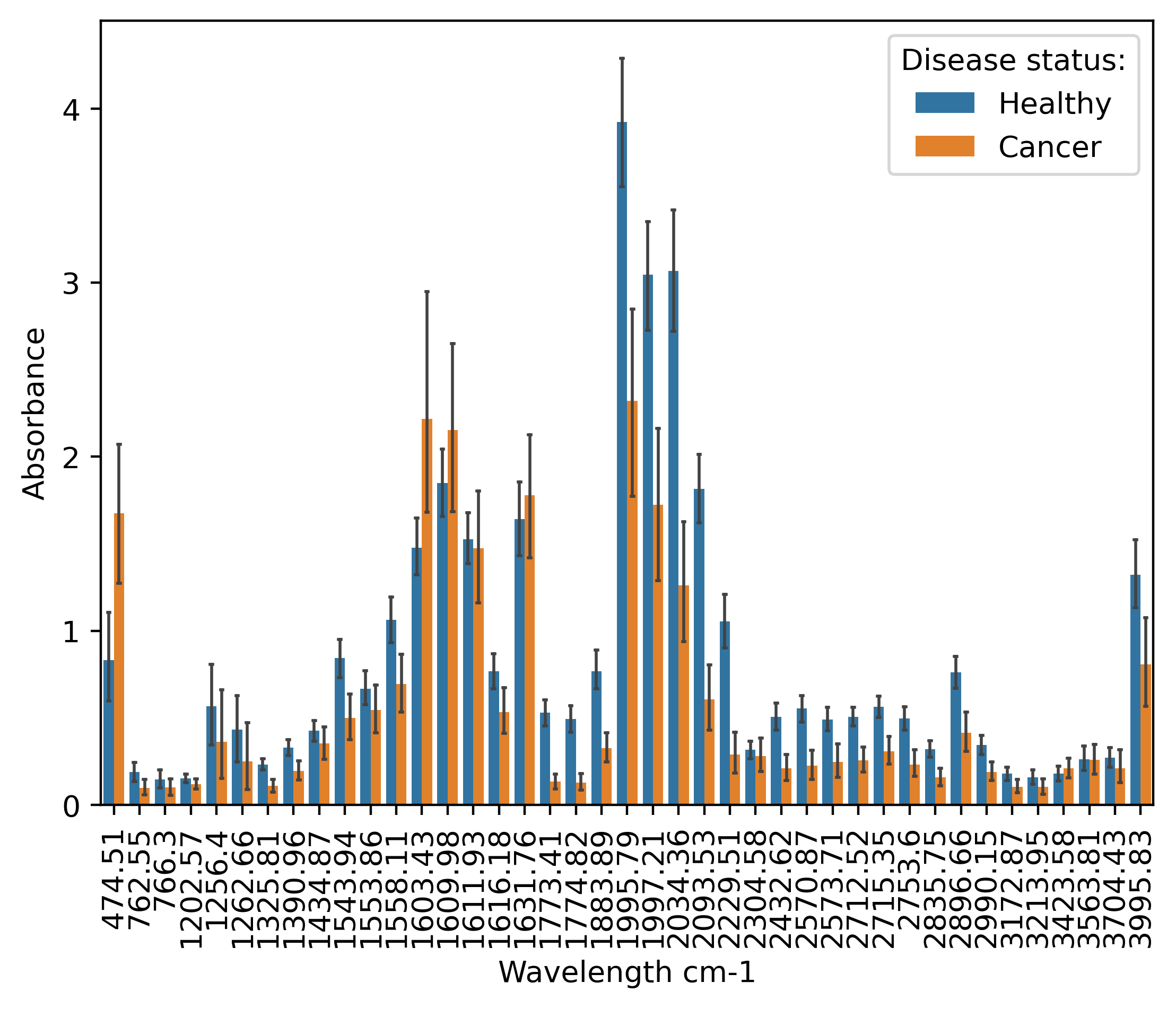

Supplement: Supplementary file 1 — In this column plot, are depicted all the frequencies that appeared to be relevant in the random forest model. (PNG 166 kb) [file 10238_2025_1684_MOESM1_ESM.png]

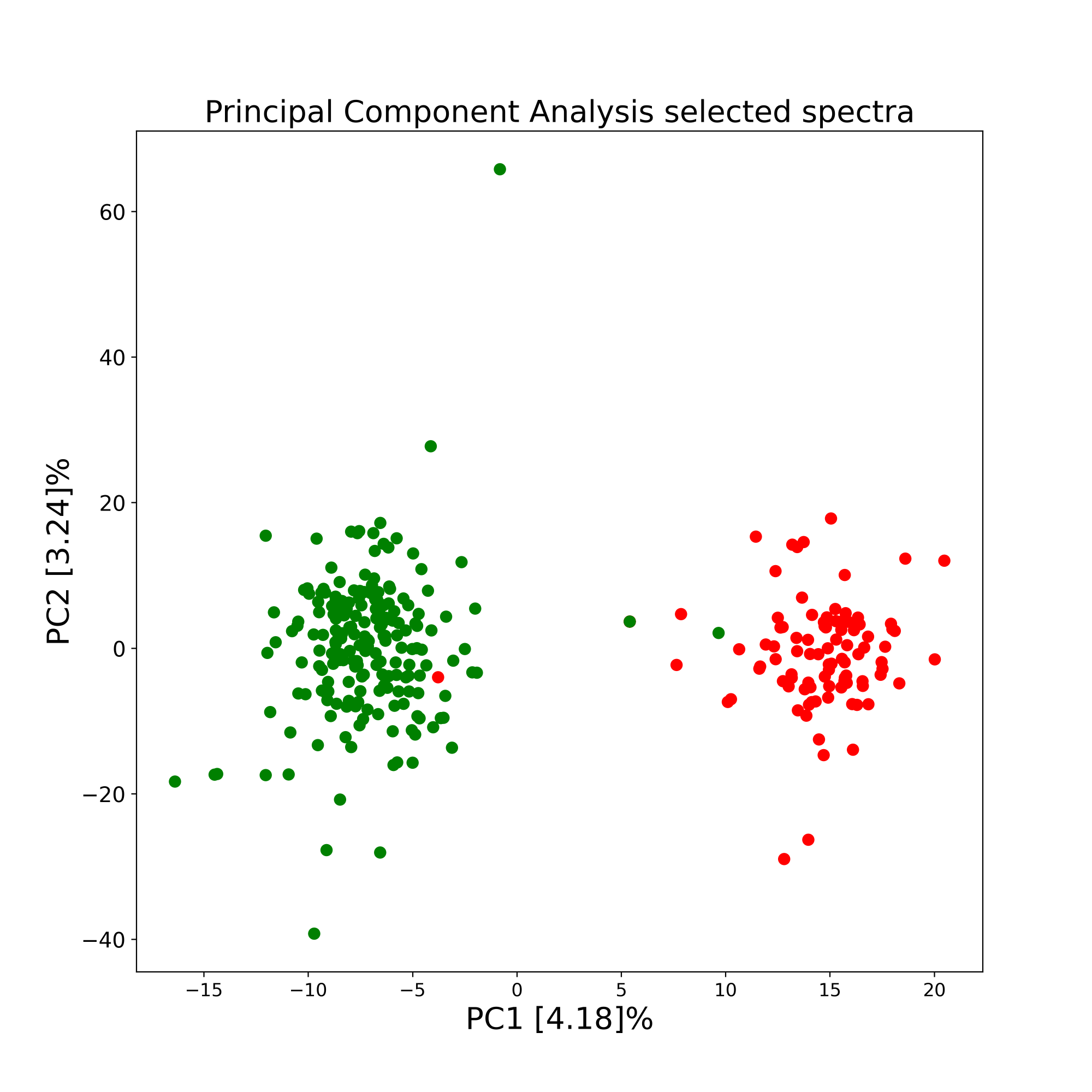

Supplement: Supplementary file 2 — PCA analysis of water removed spectra without additional data preprocessing, filtering the 46 frequencies found to be relevant from the RF classifier. (TIF 15625 kb) [file 10238_2025_1684_MOESM2_ESM.tif]

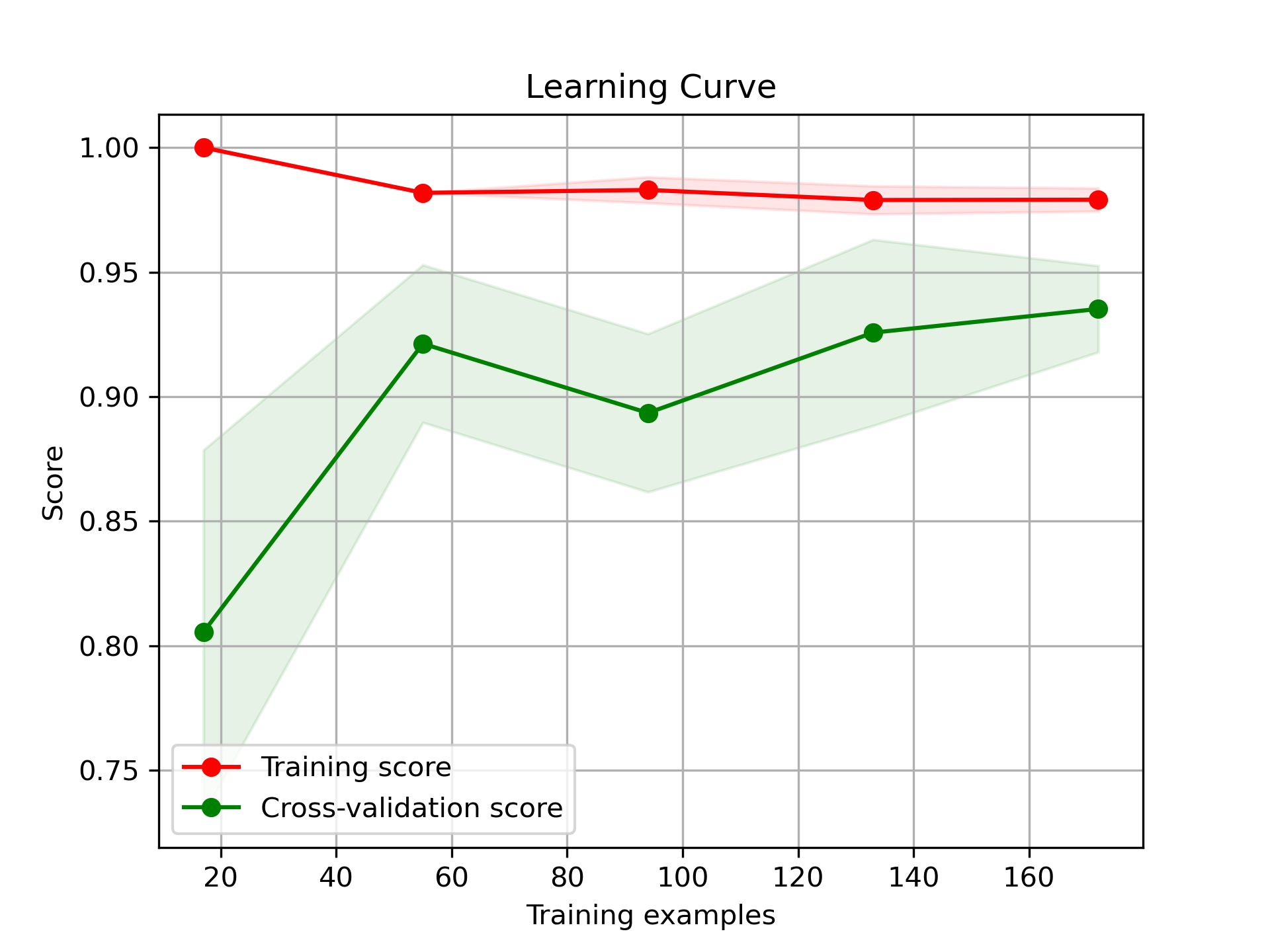

Supplement: Supplementary file 3 — Learning curve, to evaluate the performance of our model. The curve demonstrates the relationship between the training size and the model’s accuracy. It shows how the model’s performance improves as the size of the training dataset increases. The training score (in red) indicates good performance of the model, the green curve with the confidence interval shows how the model could benefit from additional unseen data. Both curves appear to converge with more training examples, indicating diminishing model improvement from additional data. The increased data fed to the model shows a reduced score, indicating that our model is not overfitting. (TIF 10800 kb) [file 10238_2025_1684_MOESM3_ESM.tif]

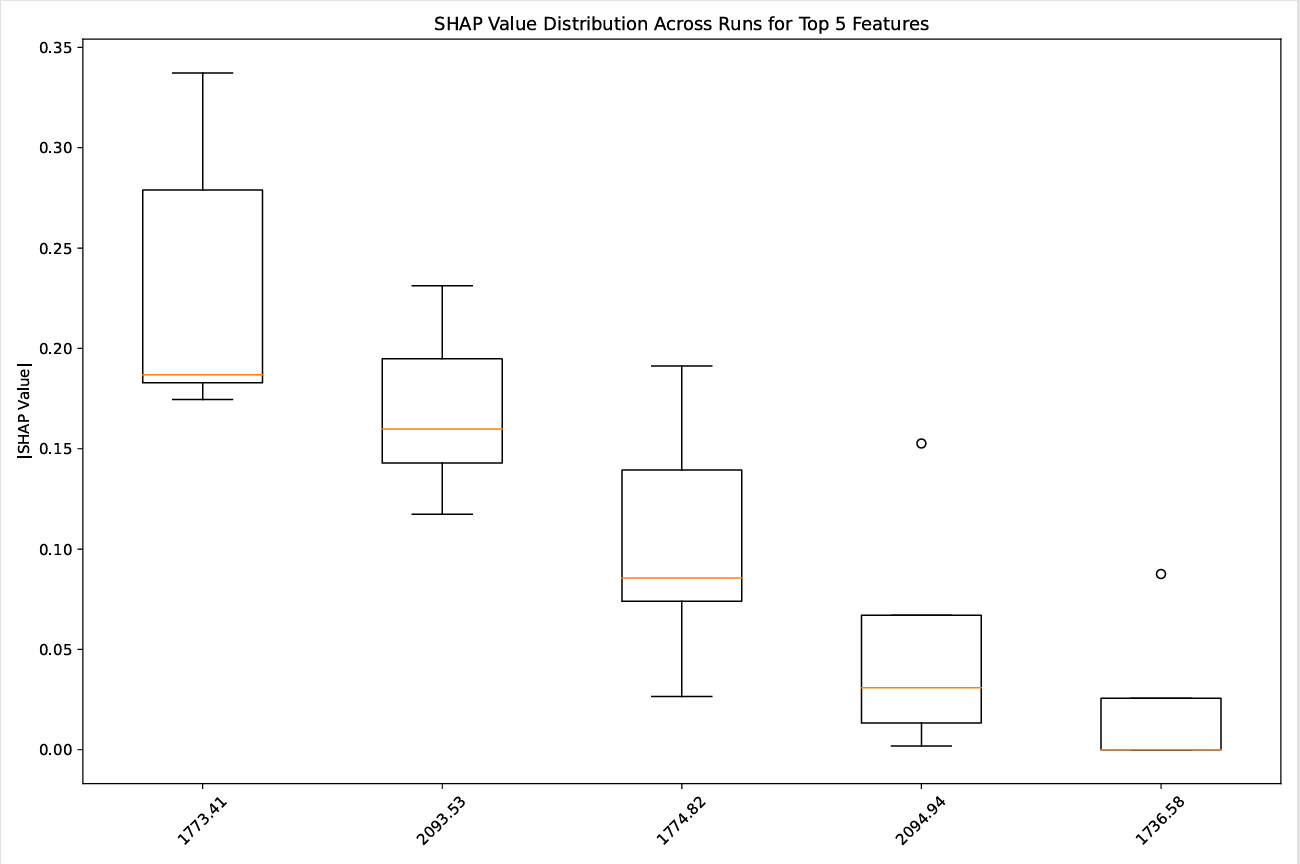

Supplement: Supplementary file 4 — We used a random seed (333) for the train/split and we handled the class imbalance using class weighting. The stability of our feature importance interpretations was assessed by analyzing SHAP value distributions across multiple independent model runs with different random seeds. As shown in the figure, the top features maintained consistent importance rankings across runs, with the most influential features (feature1 and feature2 and feature3) showing strong stability. This analysis confirms that our model's interpretations are robust to variations in data sampling and model initialization, strengthening confidence in the identified biomarkers. (PNG 24 kb) [file 10238_2025_1684_MOESM4_ESM.png]

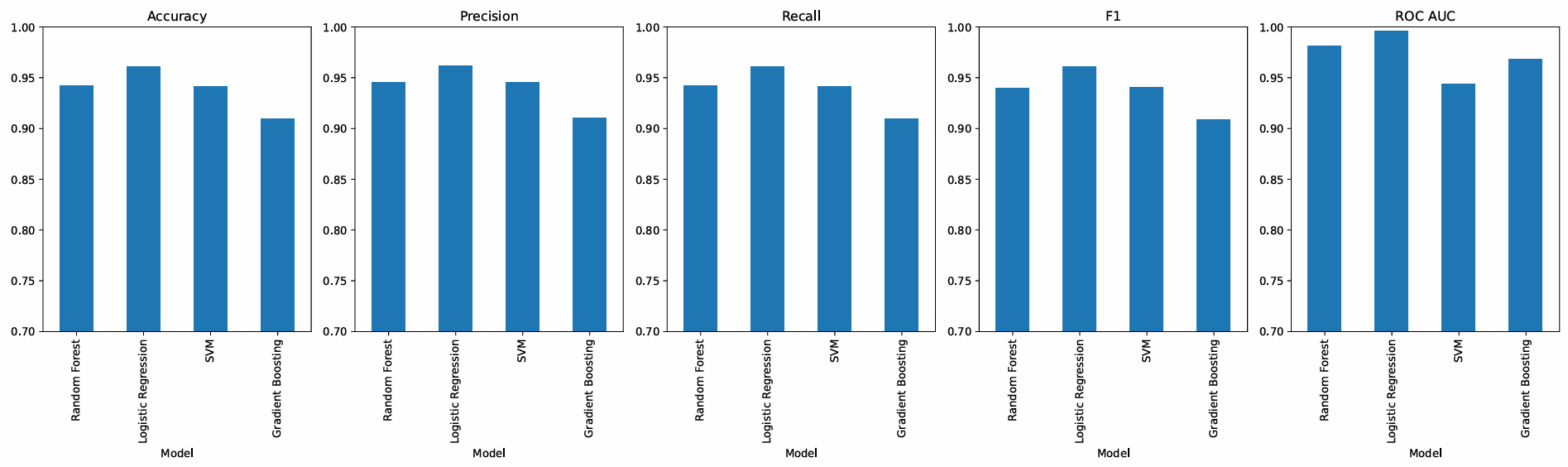

Supplement: Supplementary file 5 — For model selection, we compared Random Forest against Logistic Regression, Support Vector Machines (SVM), and Gradient Boosting using 5-fold cross-validation. As shown in Figure below, all models achieved high performance metrics, with Logistic Regression slightly outperforming others across accuracy (96%), precision (96%), recall (96%), and F1 (96%). However, we selected Random Forest for our final analysis due to its strong performance combined with its interpretability advantages through built-in feature importance measures and compatibility with SHAP for detailed feature attribution. (PNG 25 kb) [file 10238_2025_1684_MOESM5_ESM.png]
